# Supplementary material for: Complex‐centric proteome profiling by SEC‐SWATH‐MS
Source: Mol Syst Biol. 2019 Jan 14;15(1):e8438. doi: 10.15252/msb.20188438 (PMC6346213; doi:10.15252/msb.20188438)
Supplement: Supplementary file 7 — Dataset EV6 [file MSB-15-e8438-s007.zip › feature_plots_bioplex/P28065.pdf]

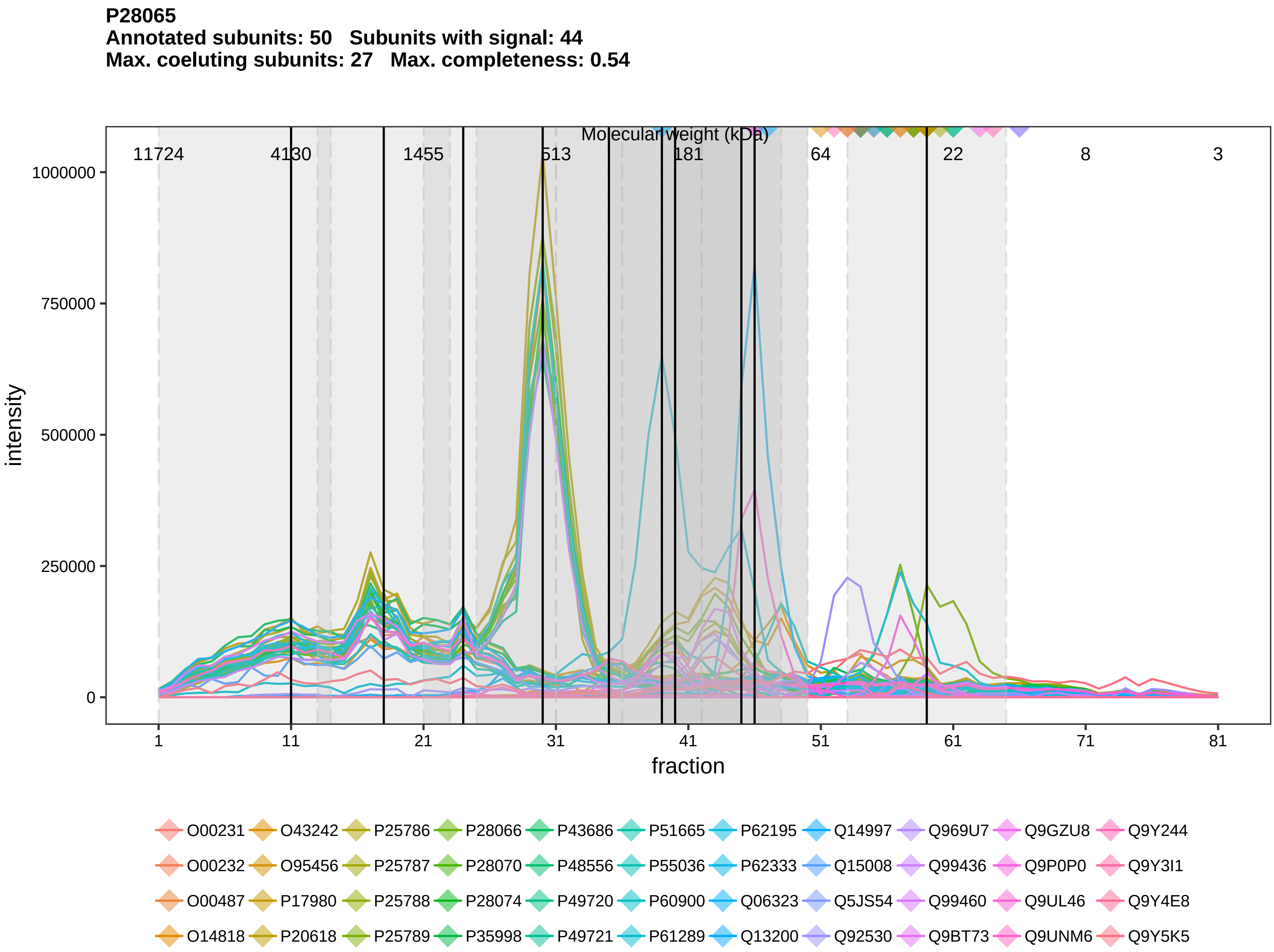
P28065
Annotated subunits: 50 Subunits with signal: 44
Max. coeluting subunits: 27 Max. completeness: 0.54
Molecular weight (kDa)
11724
4130
1455
513
181
64
22
8
3
intensity
1000000
750000
500000
250000
0
fraction
1
11
21
31
41
51
61
71
81
O00231
O00232
O00487
O14818
O43242
O95456
P17980
P20618
P25786
P25787
P25788
P25789
P28066
P28070
P28074
P35998
P43686
P48556
P49720
P49721
P51665
P55036
P60900
P61289
P62195
P62333
Q06323
Q13200
Q14997
Q15008
Q5JS54
Q92530
Q969U7
Q99436
Q99460
Q9BT73
Q9GZU8
Q9P0P0
Q9UL46
Q9UNM6
Q9Y244
Q9Y3I1
Q9Y4E8
Q9Y5K5
